# Beyond the limits of space and time: Can 4D imaging enhance postmortem investigation?

**DOI:** 10.1186/s41747-026-00806-y

**Published:** 2026-09-21

**Authors:** Dominic Gascho, Marco Palmesino, Michael Josef Thali, Edda Emanuela Guareschi

**Affiliations:** 1 Institute of Forensic Medicine, University of Zurich, Zurich, Switzerland; 2 Independent Researcher, Lugano, Switzerland; 3 South Tyrol Museum of Archaeology, Bolzano, Italy; 4 School of Medical, Molecular and Forensic Sciences, Murdoch University, Murdoch, WA Australia

**Keywords:** Autopsy, Forensic imaging, Magnetic resonance spectroscopy, Postmortem imaging, Tomography (x-ray computed)

## Abstract

**Abstract:**

Postmortem imaging has become an integral component of modern forensic pathology, with postmortem computed tomography (PMCT) and magnetic resonance (PMMR) imaging now routinely supplementing or, in selected cases, partially replacing conventional autopsy. Despite these advances, postmortem imaging remains predominantly static, capturing anatomy and injuries at a single point in time after death. In contrast, clinical radiology has increasingly embraced four-dimensional (4D) imaging approaches that incorporate time as a diagnostic dimension, enabling the assessment of motion, functional change, and dynamic processes. This narrative review with a conceptual perspective explores whether and how 4D concepts can be meaningfully translated into postmortem forensic imaging. By reviewing simulated motion models, multiphase PMCT angiography, ventilated PMCT, serial documentation of postmortem biological and physical processes, fracture mechanics research, and PMMR spectroscopy, it can be argued that time is already an implicit diagnostic dimension in forensic radiology. Although true real-time 4D imaging of the deceased remains technically limited, existing studies demonstrate that temporal reconstruction through controlled simulation and serial acquisition is feasible and forensically relevant. Embracing 4D thinking may allow postmortem imaging to evolve from static documentation toward a more process-oriented tool for reconstructing events and mechanisms leading to death.

**Key Points:**

- ***Question*** Can postmortem imaging provide time-related information that is already present after death?
- ***Findings*** 4D PMMCT and PMMR introduce time as an explicit element, allowing postmortem imaging to capture dynamic and evolving processes.
- ***Relevance statement*** This review highlights the emerging role of time-resolved (“4D”) approaches in postmortem imaging, demonstrating how integrating temporal information can enhance reconstruction of injury mechanisms, postmortem processes, and medicolegal interpretation, thereby expanding forensic radiology beyond static documentation toward process-oriented forensic investigation.

**Graphical Abstract:**

**Figure:**
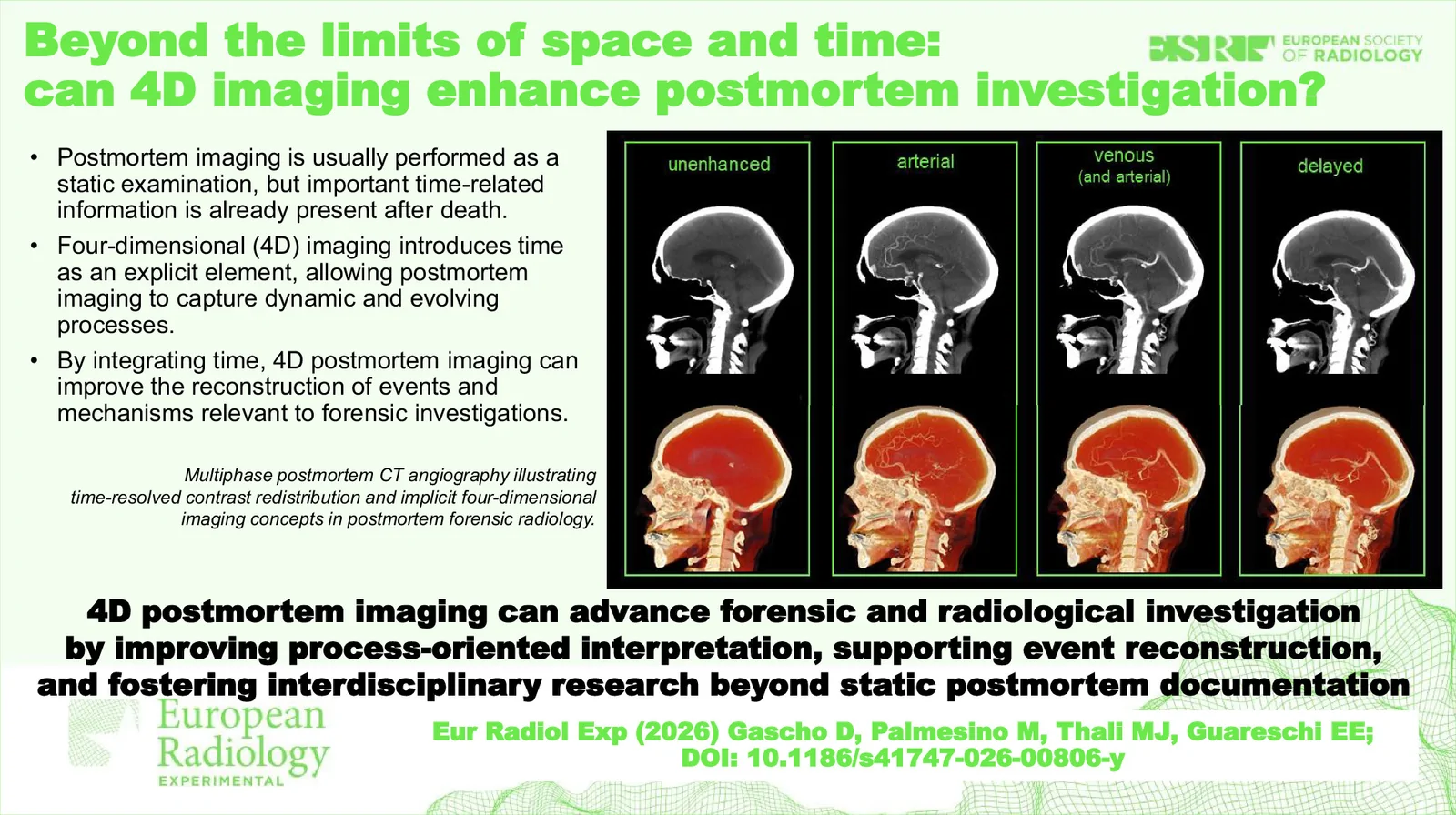

## Background

Over the past two decades, postmortem imaging has evolved from a group of complementary techniques to an increasingly integral component of forensic pathology. Virtual autopsy (also known as “virtopsy”) using postmortem computed tomography (PMCT) and postmortem magnetic resonance (PMMR) imaging is now routinely applied in many forensic institutes worldwide, particularly in the investigation of traumatic deaths, cardiovascular pathology, and cases in which traditional autopsy is limited, declined, or contested [1, 2]. These modalities provide high-resolution three-dimensional (3D) representations of the human body, allowing non-invasive visualization of skeletal injuries, gas distributions, foreign bodies, and selected soft-tissue alterations. Nevertheless, postmortem imaging remains largely static, capturing anatomy and injuries at a single point in time after death.

In parallel, clinical radiology has undergone a conceptual expansion beyond static morphology. Four-dimensional (4D) imaging introduces time as a fourth dimension, enabling visualization of movement and dynamic physiological processes. In clinical practice, 4D imaging is firmly established in cardiology, pulmonology, and radiotherapy planning, where it is used to assess cardiac motion, respiratory mechanics, vascular flow, and organ displacement [3–6]. These techniques generate time-resolved sequences of volumetric datasets, capturing changes in tissue position, density, and configuration across multiple phases.

Despite its growing relevance in clinical diagnostics, the application of 4D imaging concepts to the postmortem context remains largely unexplored. Only isolated attempts have been made to translate dynamic imaging approaches to the deceased human body. This gap is notable, given that forensic pathology is fundamentally concerned not only with documenting injuries but also with reconstructing processes, sequences of events, and mechanisms of injury. In many forensic cases, particularly those involving complex trauma, penetrating injuries, or suspected functional disturbances, understanding the dynamics of injuries can be as important as determining their presence.

At first glance, the immobility of the dead body appears fundamentally incompatible with motion-based imaging. However, movement in a forensic 4D context need not be limited to active physiological motion. Time-dependent phenomena such as externally induced tissue deformation, fluid redistribution, gas expansion, ballistic interactions, or controlled simulation of mechanical forces can all be conceptualized within a 4D framework. Furthermore, serial imaging, image-based motion modeling, and virtual reconstruction techniques allow dynamic representations to be derived from otherwise static datasets. From this perspective, 4D imaging offers the potential to extend forensic imaging beyond structural documentation toward a deeper understanding of injury dynamics and temporal relationships.

This narrative review aims to stimulate discussion on whether and how 4D imaging could emerge as a valuable diagnostic tool in postmortem forensic pathology. By discussing current technological foundations, conceptual challenges, and future research directions, postmortem 4D imaging is presented as an extension of established postmortem imaging techniques. The central question is not whether time can be reintroduced into the dead body, but whether forensic pathology is prepared to conceptualize postmortem imaging beyond the limits of static space.

## Literature search

This narrative review is based on a structured literature search performed in PubMed and Scopus to identify studies related to time-resolved concepts in postmortem imaging. Search terms included combinations of “postmortem imaging”, “4D imaging”, “PMCT”, “PMMR”, “postmortem CT angiography”, “ventilated postmortem CT”, “finite element analysis”, and “MR spectroscopy”. Articles were selected based on their relevance to dynamic or time-dependent approaches in postmortem imaging, including studies addressing simulated motion models, multiphase imaging protocols, serial imaging of postmortem changes, biomechanical modeling, and spectroscopic analysis of postmortem biochemical processes. Additional relevant publications were identified through manual screening of the reference lists of key articles. The selection focused on studies that illustrate how temporal information can be incorporated into postmortem imaging to support forensic interpretation and reconstruction of biological, physical, or biomechanical processes.

In this review, the term “4D imaging” is used in a conceptual sense to describe postmortem imaging approaches that incorporate time as an explicit analytical dimension. In contrast to conventional radiological usage, where 4D imaging typically refers to continuous time-resolved acquisition (*e.g*., cine CT or flow MRI), the term is used here as an umbrella concept for time-indexed postmortem datasets and reconstructions. This includes imaging approaches in which temporal information is derived from sequential acquisitions, multiphase imaging protocols, externally induced mechanical states, or image-based computational modeling. The purpose of this conceptual framework is not to redefine 4D imaging in a strict technical sense, but to highlight how temporal information can be integrated into postmortem imaging to support reconstruction of biological, physical, and biomechanical processes relevant to forensic investigation.

## Simulated motion and postmortem 4DCT

To date, the only study that explicitly applies a 4D imaging concept to the postmortem human body is the proof-of-concept work by Hansen et al [7]. In this study, a 4DCT model was developed using simulated closed-chest compressions in a recently deceased individual. Although primarily designed for cardiopulmonary resuscitation research, this work provides a pivotal reference point for postmortem 4D imaging. Incremental static compressions of the thorax were applied using a CT-compatible mechanical device, with volumetric CT acquisitions performed at precisely defined compression depths. These multiphase datasets were subsequently combined into time-resolved 3D sequences, effectively creating a 4D representation of thoracic and upper abdominal organ motion. Importantly, the temporal dimension was reconstructed from a series of discrete, reproducible mechanical states, conceptually comparable to stop-motion animation. The resulting models visualized skeletal deformation, cardiac displacement, vascular motion, diaphragmatic excursion, and abdominal organ movement during simulated chest compression. Even in the absence of active physiological processes, meaningful insights into internal movement patterns were obtained. From a forensic perspective, this study demonstrates that externally induced motion in a deceased body can be technically achieved and quantitatively analyzed. At the same time, the authors acknowledged important limitations, including the static nature of the incremental compressions and the influence of postmortem changes such as vascular collapse or rigor mortis.

Despite these constraints, the study by Hansen et al [7] challenges the notion that postmortem imaging must remain static. It suggests that postmortem 4D imaging should be understood as a hybrid approach, combining static volumetric acquisition with controlled mechanical or virtual simulation of motion. This paradigm is directly transferable to forensic questions involving injury reconstruction, interactions between objects and bodies, and complex sequences of force application.

## Multiphase PMCT angiography as implicit 4D imaging

Temporally resolved imaging is already an established component of forensic radiology through multiphase postmortem CT angiography (PMCTA). Standardized multiphase PMCTA protocols typically include a native (unenhanced) CT scan followed by arterial, parenchymal, and venous contrast phases [8]. Interpretation is inherently time-dependent, relying on comparison across phases rather than on a single dataset. The temporal dimension of multiphase PMCTA serves several critical purposes. Reproducibility across phases helps distinguish true pathological findings from artefacts, while dynamic contrast extravasation supports identification of bleeding sources and vascular lesions. In cases of penetrating trauma, contrast trajectories through soft tissues can assist in reconstructing wound channels and projectile paths.

**Fig. 1:**
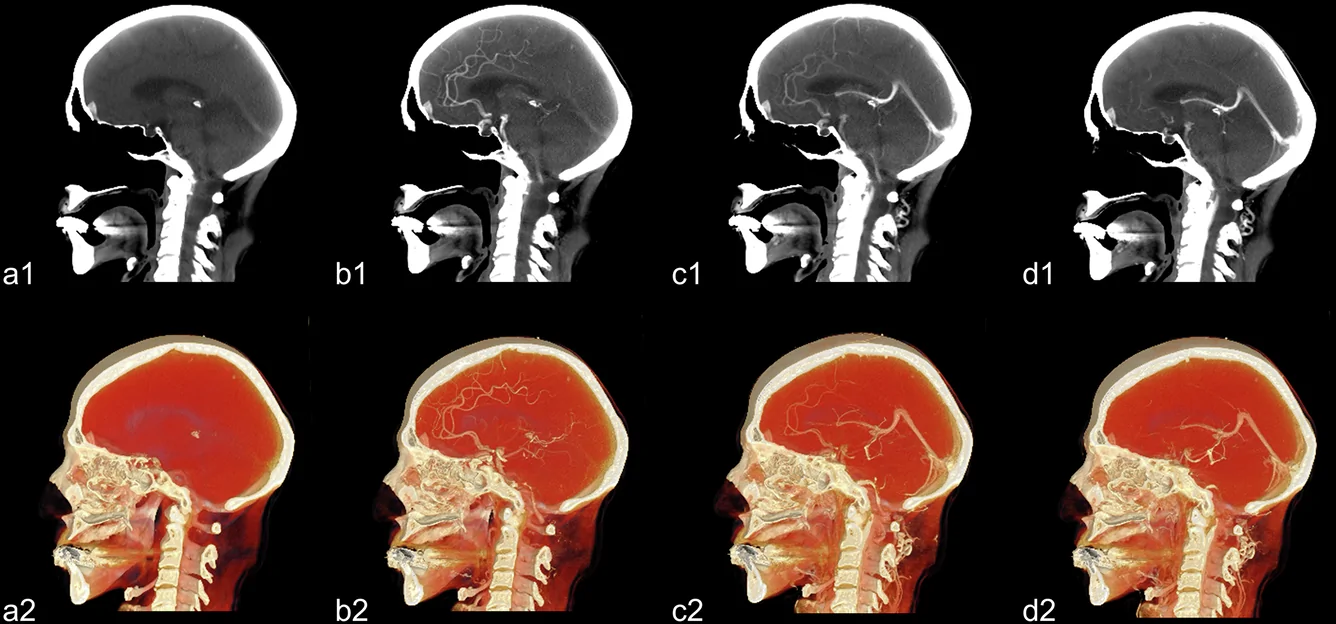
Multiplanar reconstructions (upper row) and corresponding volume rendering (lower row) of postmortem CT angiography of the head. Images are shown as native (non contrast) CT (**a1**, **a2**), arterial phase (**b1**, **b2**), venous phase (**c1**, **c2**), and a delayed acquisition 10 min after the venous phase (**d1**, **d2**). While standardized multiphase postmortem CT angiography protocols typically include multiple arterial and venous phases, the present example comprises a single arterial phase followed by venous and delayed imaging. Comparison across sequential datasets demonstrates time-dependent redistribution and persistence of intravascular contrast and allows differentiation of true vascular findings from artefacts. Even in this reduced protocol, serial postmortem angiographic acquisitions illustrate how temporal information can be reconstructed from discrete volumetric datasets, reflecting an implicit 4D imaging approach in forensic radiology

Conceptually, multiphase PMCTA can be regarded as an implicit 4D imaging technique (Fig. 1). Although it does not visualize continuous motion, it provides a sequence of 3D datasets acquired at defined time points, allowing assessment of temporal changes in vascular filling and contrast distribution. The widespread acceptance of multiphase PMCTA demonstrates that forensic pathology is already prepared to integrate time as a diagnostic dimension, provided that protocols are standardized and interpretation frameworks are established. In this sense, explicit postmortem 4D imaging represents an extension rather than a paradigm shift.

## Ventilated PMCT as functional 4D imaging

Ventilated postmortem CT represents another example of functional, time-resolved postmortem imaging. Developed to overcome diagnostic limitations caused by postmortem hypostasis and atelectasis, ventilated PMCT mimics clinical inspiratory CT by artificially inflating the lungs after death [9].

Serial CT acquisitions before and during ventilation generate a temporal sequence in which lung volume, gas distribution, and attenuation patterns evolve. Studies have shown that increasing ventilation pressures result in progressive lung expansion, displacement of the heart, and partial reversal of dependent lung opacities [9]. In traumatic cases, serial ventilation has been used to evaluate pneumothoraces, hemothoraces, and wound channels, with imaging findings changing across successive scans.

From a 4D perspective, ventilated PMCT visualizes pressure-dependent temporal change rather than spontaneous biological motion. It demonstrates that functional imaging after death is feasible and that controlled external intervention can generate diagnostically meaningful dynamic datasets.

## Fracture mechanics and the limits of postmortem 4D imaging

The visualization of short-term force application and fracture initiation represents the most challenging temporal scale for postmortem 4D imaging. Experimental biomechanics has demonstrated that a fracture is a dynamic process. High-speed x-ray imaging has captured real-time fracture initiation and propagation during simulated falls in cadaveric specimens [10], while synchrotron-based micro-CT studies have resolved crack propagation and strain evolution under incremental loading [11].

Direct translation of these techniques into routine forensic practice is currently unrealistic due to technical and logistical constraints. Nevertheless, incremental loading experiments combined with sequential volumetric imaging provide a feasible intermediate strategy. Even without capturing the precise moment of failure, time-resolved imaging across controlled mechanical states can support differentiation of primary and secondary fractures and improve biomechanical reconstruction of injury sequences.

In addition, PMCT datasets can serve as the geometric and material basis for biomechanical modeling approaches such as finite element analysis [12, 13]. Subject-specific finite element models derived from postmortem imaging allow simulation of time-dependent stress distribution, deformation, and fracture initiation under defined loading conditions. Although such models do not constitute imaging in the strict sense, they generate dynamic representations of biomechanical processes grounded in image-based anatomy. In this broader conceptual framework, image-based biomechanical simulation may be regarded as an indirect form of postmortem 4D imaging, in which temporal evolution is reconstructed computationally rather than acquired experimentally.

## Time-resolved imaging of postmortem biological and physical processes

The systematic documentation of biological and physical changes after death can be conceptualized through 4D postmortem imaging. Repeated PMCT or PMMR under standardized conditions allows visualization of time-dependent alterations in tissue density, organ volume, and gas distribution.

**Fig. 2:**
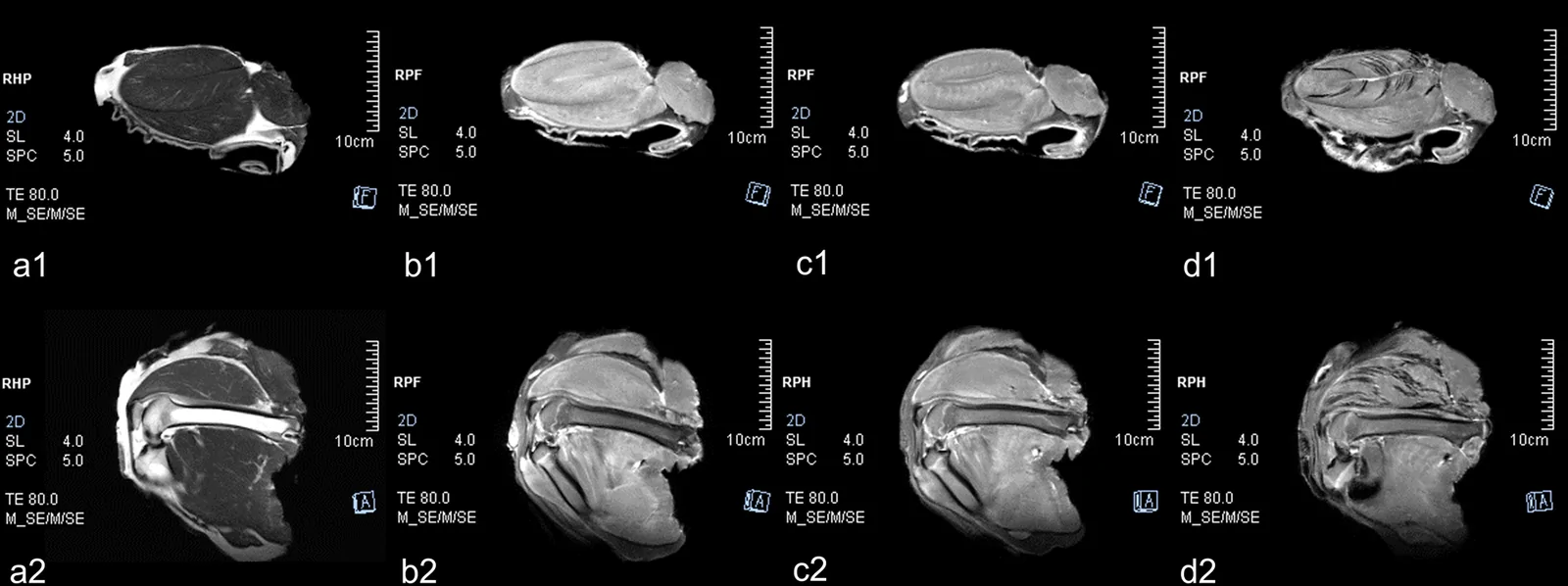
Time-resolved postmortem MRI of a sheep hind limb illustrating 4D concepts through serial acquisition. T2-weighted images are shown in coronal (upper row) and sagittal (lower row) orientation at increasing postmortem intervals: approximately 8 h (**a1**, **a2**), 29 h (**b1**, **b2**), 57 h (**c1**, **c2**), and 80 h (**d1**, **d2**). The pronounced image contrast differences in the initial dataset (**a**) are predominantly attributable to temperature-related effects shortly after death, whereas from **b1**, **b2** to **d1**, **d2**, the specimens were stored under controlled cooling conditions. In the final time point (**d1**, **d2**), intramuscular gas formation aligned with muscle fiber architecture is visible, consistent with advanced postmortem decomposition. This longitudinal MRI approach demonstrates how temporal information can be reconstructed from discrete volumetric datasets, constituting an implicit form of 4D imaging in postmortem forensic radiology. Representative anatomical PMMR images acquired during the experimental MR spectroscopy study by Gascho et al [16]. The figure was created specifically for this review; the MRI images shown were not part of the original publication

Klein et al [14] performed whole-body PMCT at hourly intervals for up to 36 h, documenting progressive changes in cerebrospinal fluid density, lung and liver volumes, and bowel distension. Time-lapse reconstructions effectively created 4D representations of early postmortem physiology. Similarly, Bolliger et al [15] demonstrated that serial CT and MR imaging during thawing of frozen cerebral tissue reveal characteristic, time-dependent imaging artefacts that allow reliable differentiation from putrefactive gas. Extending this concept to tissue microstructure and composition, Gascho et al [16] showed that serial PMMR imaging and spectroscopy of muscle and bone marrow capture reproducible, time-dependent signal alterations related to biochemical degradation processes, independent of mechanical motion (Fig. 2). These studies fulfill the core criteria of 4D imaging by combining volumetric data with an explicit temporal axis. Although no mechanical motion is visualized, dynamic transformation of tissue properties is captured in a manner highly relevant to forensic interpretation, including differentiation of ante-mortem pathology from postmortem change and refinement of postmortem interval estimation. Beyond conventional PMMR, emerging multinuclear MRI techniques further illustrate the potential of quantitative postmortem imaging. Recent experimental work in a postmortem porcine model demonstrated that ^23^Na MRI enables non-invasive quantification of vitreous sodium concentrations and their time-dependent alteration after immersion in fresh- and saltwater, illustrating how advanced PMMR techniques can capture dynamic biochemical processes relevant to forensic investigations [17].

## Postmortem MR spectroscopy as biochemical 4D imaging

Temporal information in postmortem imaging is not limited to morphology or mechanics. Postmortem MR spectroscopy (PMMRS) represents a biochemical form of 4D imaging in which time is encoded in metabolite composition rather than spatial displacement. In contrast to morphologic imaging, which captures structural change, spectroscopy reflects underlying biochemical processes that evolve predictably after death.

Ith et al [18] demonstrated that serial *in situ* ¹H PMMRS of decomposing brain tissue reveals systematic and reproducible temporal patterns. Early postmortem phases are dominated by the decay of physiological metabolites such as N-acetylaspartate, creatine, and choline-containing compounds. Approximately 3 days after death, additional metabolites that are absent *in vivo* begin to appear, including free trimethylammonium, propionate, butyrate, and iso-butyrate. These changes reflect the transition from autolysis to bacterial decomposition and define a biologically meaningful temporal boundary. Building on this metabolic characterization, Scheurer et al [19] introduced a quantitative statistical framework in which time-dependent metabolite concentration curves were modeled mathematically. By inverting these functions, the postmortem interval could be estimated from a single spectroscopic measurement with defined confidence intervals. This approach marked a conceptual shift from descriptive postmortem change toward predictive, model-based interpretation. Time is no longer observed directly but inferred from a multidimensional biochemical state. Subsequent work by Ith et al [20] demonstrated that ambient temperature exerts a systematic influence on postmortem metabolic trajectories. By incorporating temperature as an explicit parameter, the authors showed that metabolite concentration changes follow predictable functions until the onset of advanced bacterial decomposition. This parametric approach effectively adds an environmental dimension to postmortem 4D imaging and underscores that time in the postmortem context cannot be interpreted independently of external conditions.

This parametric approach effectively adds an environmental dimension to postmortem 4D imaging and underscores that time in the postmortem context cannot be interpreted independently of external conditions. This concept was further advanced by Zoelch et al [21], who demonstrated that PMMRS can be used for *in situ* temperature determination in postmortem brain tissue over a wide range of postmortem intervals. By applying PMMRS thermometry with lactate as a temperature-stable reference metabolite, the authors showed that local brain temperature can be measured noninvasively with high accuracy even in advanced postmortem states. Beyond its practical relevance for protocol adjustment and temperature correction in PMMR and PMMRS, this work highlights temperature as a measurable and quantifiable parameter rather than an external assumption. In the context of postmortem 4D imaging, PMMRS-based thermometry therefore provides a means to directly integrate environmental temperature into temporal models of postmortem change, reinforcing the notion that biochemical and physical time axes are inseparably linked.

In summary, PMMRS represents a unique biochemical and thermophysical extension of 4D imaging. It complements morphologic and mechanical models by providing access to metabolic and temperature-dependent time markers that are largely independent of macroscopic structural change. While not yet part of routine forensic practice, this approach illustrates how 4D thinking can extend beyond spatial imaging and contribute to objective, quantitative assessment of postmortem processes under defined environmental conditions.

## Integration of 4D concepts into virtopsy

The rise of 4D postmortem imaging concepts must be viewed within the broader framework of virtual autopsy. Virtual autopsy has become established as a minimally invasive alternative or ancillary to conventional autopsy, particularly in contexts where cultural, religious, or logistical constraints limit invasive procedures [1]. To this end, postmortem imaging has traditionally focused on static documentation. However, the integration of time-resolved approaches offers substantial added value. In virtual autopsy, 4D concepts can enhance reconstruction of perimortem events, clarify injury mechanisms, and support differentiation between ante-mortem pathology and postmortem alterations. Serial imaging protocols, simulated motion models, and multiphase contrast techniques already demonstrate that temporal information can be extracted even in the absence of physiological activity. Table 1 summarizes the principal approaches discussed in this review and illustrates how temporal information can be integrated into postmortem imaging across different methodological frameworks. To enhance interpretability, the approaches are additionally categorized according to their level of forensic maturity and validation. This classification is informed by the current literature and reflects the heterogeneous level of validation and implementation across techniques, including recent comprehensive reviews on the state of forensic imaging and guideline-based recommendations, as well as methodological and application-oriented reviews addressing specific techniques such as postmortem MR spectroscopy [1, 17, 22, 23].

**Table 1:** Conceptual framework of postmortem 4D imaging

Technique	Time scale	What changes	Forensic question	Forensic maturity/level of validation
Multiphase PMCTA	Seconds–minutes	Contrast distribution	Bleeding source	Established technique, routine use in specialized centers
Ventilated PMCT	Minutes	Lung volume, gas distribution	Pneumothorax	Emerging application, limited routine use
Serial PMCT/PMMR	Hours–days	Density, gas formation	Postmortem interval	Emerging application, primarily research and selected casework
PMMRS	Hours–days	Metabolite composition	Postmortem interval	Experimental to emerging, research-based
External deformation CT	Seconds	Organ displacement	Trauma mechanics	Proof-of-concept, experimental
Finite element modeling	Simulated	Stress distribution	Fracture sequence	Experimental, research-stage

*CT* Computed tomography, *PMCT* Postmortem computed tomography, *PMCTA* Postmortem computed tomography angiography, *PMI* Postmortem interval, *PMMR* Postmortem magnetic resonance imaging, *PMMRS* Postmortem magnetic resonance spectroscopy

## From implicit temporal data to process-oriented forensic interpretation

The preceding sections illustrate that 4D concepts are already embedded, albeit implicitly and heterogeneously, within contemporary postmortem forensic imaging. Rather than representing a single, clearly defined technique, postmortem 4D imaging currently manifests as a spectrum of approaches that integrate time through simulation, serial acquisition, functional intervention, or biochemical modeling. This diversity reflects both the interdisciplinary nature of forensic radiology and the wide range of temporal scales relevant to medicolegal questions.

A central strength of 4D approaches in postmortem imaging lies in its alignment with the core objectives of forensic pathology. Forensic investigations rarely focus solely on the presence or absence of anatomical findings; instead, they seek to reconstruct processes, mechanisms, and sequences of events. Static imaging, while invaluable for documentation, often provides limited insight into causality. By contrast, time-resolved imaging, whether through multiphase angiography, ventilated CT, or simulated motion models, explicitly addresses how findings may have evolved or interacted. This stepwise approach is particularly well-suited to forensic practice, where ethical, technical, and logistical constraints often preclude continuous imaging.

Nevertheless, significant challenges remain. Technical limitations of clinical CT and MRI systems restrict temporal resolution, making the direct visualization of rapid events, such as fracture initiation or ballistic interactions, largely unattainable in routine casework. Data management and interpretation also pose challenges, as time-resolved imaging generates large, complex datasets that require advanced post-processing, segmentation, and visualization tools. Moreover, standardized protocols and validated interpretation criteria are essential to ensure reproducibility and medicolegal robustness. Biological variability represents an additional limitation. Postmortem tissue behavior is influenced by numerous factors, including postmortem interval, temperature, cause of death, and prior medical interventions. While experimental models and controlled simulations can reduce variability, translation to individual casework must be approached cautiously. This underscores the need for interdisciplinary collaboration and systematic research to define the boundaries within which postmortem 4D imaging can provide reliable information.

Despite these challenges, the reviewed approaches highlight several promising future directions. Advances in image-based motion modeling, finite element analysis, and artificial intelligence may allow increasingly sophisticated reconstruction of dynamic processes from static or semi-dynamic datasets. Integration of biomechanical modeling with postmortem imaging could enhance reconstruction of force application, injury sequencing, and fracture mechanisms. Emerging CT technologies may further expand these possibilities. For example, photon-counting CT enables energy-resolved acquisition with improved material characterization and may facilitate more sophisticated analysis of foreign bodies, ballistic materials, and tissue composition, thereby complementing future process-oriented postmortem imaging approaches [23, 24]. Similarly, the combination of morphological 4D imaging with biochemical time markers, such as PMMRS, offers a multidimensional perspective on postmortem change that extends beyond purely structural analysis. Crucially, the development of postmortem 4D imaging should not be viewed as competition with traditional autopsy. Instead, it represents a complementary extension of forensic diagnostics, providing additional layers of interpretation that can guide, refine, or contextualize autopsy findings. At its current stage, the primary strength of postmortem 4D imaging lies in research and in the reconstruction of events and injury mechanisms, rather than in routine casework. In cases where conventional autopsy is limited or declined, time-resolved imaging may nevertheless offer unique insights that would otherwise remain inaccessible.

## Responsible use of 4D postmortem imaging

While 4D concepts offer substantial potential for postmortem forensic imaging, their application must be framed by clear methodological and ethical boundaries. Time-resolved postmortem imaging relies heavily on controlled simulation, serial acquisition, or model-based inference. As a result, findings are inherently dependent on experimental conditions, imaging protocols, and assumptions embedded within the applied models. Without careful standardization, there is a risk that dynamic reconstructions may be overinterpreted or misapplied in medicolegal contexts.

Biological variability represents a central limitation. Postmortem tissue behavior is influenced by numerous interacting factors, including cause of death, postmortem interval, ambient temperature, prior medical interventions, and body handling. Therefore, 4D imaging approaches must be interpreted as probabilistic and contextual rather than deterministic representations of perimortem events. Transparent reporting of acquisition parameters, environmental conditions, and modeling assumptions is essential to ensure forensic robustness and courtroom defensibility. Responsible use of 4D postmortem imaging also requires clear communication of its scope and limits to non-radiological stakeholders, including forensic pathologists, legal professionals, and courts. Dynamic visualizations can be intuitively compelling, but they do not constitute direct recordings of events. Instead, they represent reconstructions based on defined premises. Maintaining this distinction is crucial to prevent undue evidentiary weight being attributed to visually persuasive but model-dependent results.

## From 4D to “4W”

Postmortem forensic imaging represents a multidisciplinary diagnostic tool that supports legal authorities in addressing the fundamental forensic questions of Who, When, Where, and hoW, referred to here as the “4W” framework. This terminology represents a forensic adaptation of the well-established investigative “5W1H” approach, in which the question of what is considered implicit in the documented findings. In the forensic context, the question of why is primarily addressed through complementary forensic disciplines, while postmortem imaging contributes predominantly to the remaining dimensions through objective documentation and reconstruction.

Temporal reasoning has always been inherent to forensic investigations, including the application of postmortem imaging, where timeline analysis forms an essential interpretative component. As discussed throughout this article, postmortem imaging modalities are already implicitly time-resolved. Conceptually, the time zero reference in forensic pathology corresponds to the moment of death, but in practice, it is commonly inferred from early postmortem changes, such as the initial stages of decomposition, which therefore function as an implicit temporal anchor in imaging interpretation. By contrast, 4D postmortem imaging incorporates time explicitly and thus has the potential to provide added forensic value, either as a standalone approach or in combination with established postmortem imaging techniques. Standardized acquisition protocols and robust interpretation frameworks are essential in this context. For 4D imaging to emerge as a meaningful diagnostic tool in postmortem forensic pathology and to contribute reliably to answering the “4W” questions, it must be integrated into the same probabilistic logic that governs contemporary postmortem imaging investigations. This entails assigning higher or lower probabilities to forensic hypotheses by confirming or excluding findings through independent data sources, including other imaging modalities and information derived from the broader multidisciplinary forensic context. The diagnostic benefit of combining complementary postmortem imaging techniques has already been demonstrated, for example, through the combined use of PMCT and PMMR imaging for the detection of pulmonary thromboembolism and for detailed soft-tissue evaluation in traumatic injuries [24–27]. The forensic relevance of integrating additional forms of 4D postmortem imaging with established and scientifically validated techniques will require continued systematic evaluation and may ultimately lead to the identification of novel forensic patterns or imaging “fingerprints.” Such developments have the potential to simplify highly complex forensic scenarios and to refine answers to the fundamental “4W” questions posed by investigative authorities, thereby supporting accurate reconstruction and the pursuit of justice.

## Conclusions

Postmortem forensic imaging has traditionally been defined by static documentation of anatomy after death. While true real-time imaging of dynamic events remains technically limited, existing proof-of-concept studies demonstrate that meaningful temporal reconstruction is achievable through controlled simulation, serial acquisition, and model-based analysis. Looking forward, the continued development of postmortem 4D imaging will depend on interdisciplinary collaboration between forensic pathology, radiology, biomechanics, imaging science, and computational modeling. From a forensic perspective, the integration of time-resolved approaches into postmortem imaging has several potential applications. Temporal information may assist in reconstructing injury mechanisms, differentiating ante-mortem pathology from postmortem alterations, and improving the interpretation of dynamic processes such as hemorrhage, gas redistribution, or tissue degradation. In complex trauma cases, simulated motion models and biomechanical simulations may contribute to the reconstruction of force application and fracture sequences, while serial imaging and spectroscopic approaches may support the assessment of postmortem changes and postmortem interval estimation. Although many of these techniques are currently primarily used in experimental or research settings, they illustrate how incorporating temporal context may expand the role of postmortem imaging from static documentation toward a more process-oriented tool for forensic reconstruction. As imaging technology and analytical methods continue to advance, 4D approaches have the potential to transform postmortem imaging from static documentation into a process-oriented diagnostic tool. By explicitly embracing time alongside space, postmortem imaging can evolve from static documentation toward a process-oriented diagnostic framework that more directly supports medicolegal reconstruction and the systematic answering of the fundamental forensic “4W” questions, while fostering scientific understanding and interdisciplinary exchange within the expanding field of postmortem imaging.

## Abbreviations

3D: Three-dimensional
4D: Four-dimensional
4DCT: Four-dimensional computed tomography
CT: Computed tomography
MR: Magnetic resonance
MRI: Magnetic resonance imaging
PMCT: Postmortem computed tomography
PMCTA: Postmortem computed tomography angiography
PMMR: Postmortem magnetic resonance
PMMRS: Postmortem magnetic resonance spectroscopy
